# Necrotizing Pneumonia in Children: Early Recognition and Management

**DOI:** 10.3390/jcm12062256

**Published:** 2023-03-14

**Authors:** Yuanyuan Chen, Lanxin Li, Chenlu Wang, Yuanyuan Zhang, Yunlian Zhou

**Affiliations:** 1 Department of Pulmonology, Children’s Hospital, Zhejiang University School of Medicine, Hangzhou 310052, China; 2 National Clinical Research Center for Child Health, Hangzhou 310052, China

**Keywords:** necrotizing pneumonia, prediction, treatment

## Abstract

Necrotizing pneumonia (NP) is an uncommon complicated pneumonia with an increasing incidence. Early recognition and timely management can bring excellent outcomes. The diagnosis of NP depends on chest computed tomography, which has radiation damage and may miss the optimal treatment time. The present review aimed to elaborate on the reported predictors for NP. The possible pathogenesis of *Streptococcus pneumoniae*, *Staphylococcus aureus*, *Mycoplasma pneumoniae* and coinfection, clinical manifestations and management were also discussed. Although there is still a long way for these predictors to be used in clinical, it is necessary to investigate early predictors for NP in children.

## 1. Introduction

Necrotizing pneumonia (NP) is a rare complication of community-acquired pneumonia (CAP) and is characterized by catastrophic illness and a prolonged hospital stay and disease course [1]. It has been reported that 3% of cases of CAP in the UK have complications. Although NP alone and those co-existing with one or more other forms of complications of CAP (including parapneumonic effusion, empyema, lung abscess and local complications) are still deemed to be rare, the incidence of pediatric NP has increased [2]. A French study found that the proportion of NP among hospitalized CAP was 4.5% from 2006 to 2009, and the rate doubled to 9% from 2009 to 2011 [3]. In the United States, a retrospective observational study including 80 cases of NP identified no cases in the period 1990–1993, 12 in the period 1993–1996 and 40 in the period 2001–2004 [4].

NP usually progresses rapidly in previously healthy children despite appropriate management. A study in Taiwan found that no underlying diseases may predict the occurrence of necrosis and/or abscess independently [5]. However, a recent study has shown that NP tended to occur in children with a complex chronic condition, and the mortality rate was higher than that of NP in previously healthy children [6]. NP is characterized by necrosis and liquification of lung parenchyma and loss of the normal pulmonary parenchymal architecture [7]. Moreover, NP is associated with a higher risk of complications such as parapneumonic effusion, pleural empyema, pyothorax, pneumothorax, pyopneumothorax, septic shock, respiratory failure, hemolytic uremic syndrome (HUS) and bronchopleural fistula (BPF) [8,9]. In view of the severity of NP and heavy burden of hospitalization especially in developing countries, early prediction and management are essential for good recovery and prognosis.

## 2. Etiology and Pathogenesis

*Streptococcus pneumoniae*, *Staphylococcus aureus* and *Mycoplasma pneumoniae* are the most common pathogens reported in children with NP. Other bacterial infections associated with NP in children include *Pseudomonas aeruginosa*, *Streptococcus mitis* spp., *Streptococcus pyogenes*, *Klebsiella pneumoniae*, *Pseudomonas* spp., *Fusobacterium* spp., *Staphylococcus epidermidis*, *Enterobacter aerogenes*, *Burkholderia pickettii biovar*, *Mycobacterium tuberculosis*, and *Chromobacterium violaceum* [10,11,12]. Some viruses and fungi like influenza, parainfluenza, adenovirus, respiratory syncytial virus, Herpes group (including Cytomegalovirus, Varicella-Zoster and the Epstein–Barr Virus), *Aspergillus* spp., *Candida* spp., *Histoplasma capsulatum*, *Coccidioides* spp., *Blastomyces* spp. and *Cryptococcus neoformans* can also cause NP [6,10]. And a synergy between viruses and bacteria is common and sometimes lethal. In order to recognize earlier and develop novel therapeutic strategies, it is essential to understand the pathogenesis of NP.

### 2.1. Streptococcus pneumoniae (Pneumococcus)

Pneumococcus is a Gram-positive bacterium and is a leading cause of bacterial pneumonia in children worldwide. In 2000, it was reported that 741,000 children less than 5 years old (accounting for 36% of all-cause pneumonia deaths) succumb to pneumococcal pneumonia, with the majority of them from developing countries [13]. Necrotizing pneumococcal pneumonia in children was first reported in 1994 [14], and an increase in its prevalence has been observed since then. However, the detailed mechanism is still unknown.

The capsule polysaccharide is the major virulence factor. It assists pneumococcus in interacting with the epithelium, avoiding entrapment by nasal mucus and inhibiting opsonophagocytosis during infections [15,16]. According to structurally and antigenically different capsular polysaccharides, more than 100 serotypes have been identified until now. Pneumococcal conjugated vaccines (PCVs) bind the capsular polysaccharides to a carrier protein. It can promote the immune system of young children to produce an adequate response by enhancing immunogenicity [17]. In 2000, the 7-valent pneumococcal conjugated vaccine (PCV-7) protecting against seven serotypes (serotypes 4, 6B, 9V, 14, 18C, 19F and 23F) was first licensed in the United States [18]. Beginning in 2010, PCV-10 containing additional serotypes 1, 5, 7F and PCV-13 containing additional serotypes 1, 5, 7F, 3, 6A and 19A have become available for use [19]. The introduction of PCV-7 led to significant decreases in pneumonia in childhood but increased the incidence of NP caused by non-vaccine serotypes [20]. PCV-13 has been reported to reduce the incidence and hospitalization rates for empyema and parapneumonic effusion substantially without serotype shift [21,22]. Similarly, PCV-13 implementation decreased the hospitalization rate for NP in Italy significantly. However, the hospitalization rate increased during the late post-PCV-13 period, and serotype 3 was prevalent in both the pre- and post-PCV-13 periods, which confirmed the modest efficacy of PCV-13 against serotype 3 [23]. Serotypes 3 and 19A are associated with most necrotizing pneumococcal pneumonia [2] (Table 1).

In addition to the polysaccharide capsule, pneumolysin is another virulence factor that can breach the membrane barrier of cells as a pore-forming toxin. In vitro, respiratory epithelial cells can sense the osmotic stress of pore formation and then activate the p38 mitogen-activated-protein kinase [25]. The activation of mitogen-activated-protein kinase may lead to an increase in chemokines and neutrophil influx [26]. Pneumolysin may also activate complement and inhibit neutrophil respiratory burst and release of the antibacterial, vasodilatory and nitric oxide from macrophages [27]. In the later phases of lung infection, pneumolysin is released from the cytoplasm to the surrounding tissue by the lysis of pneumococcus caused by neutrophils or activation of Lyt A [28]. Then this may lead to widespread damage to host tissue and help pneumococcus survive. Nevertheless, the increased virulence contributed by pneumolysin is not universal. For example, serotype 1 ST306, which is associated with invasive pneumococcal diseases, produces nonhemolytic pneumolysin [29].

Pathologic examinations have shown that suppurative necrosis with extensive infiltration and accumulation of neutrophils in the lung parenchyma and coagulative necrosis with anucleated and eosinophilic cell contours in preserved outlines of lung architecture is observed in children with NP [30]. Therefore, cytokines that promote neutrophilic inflammation and activate coagulation may be associated with the severity of NP. For example, IL-8 can effectively recruit and activate neutrophils, and the level of IL-8 has been reported to be related to the severity of lung necrosis [31].

### 2.2. Staphylococcus aureus

*Staphylococcus aureus* is a Gram-positive, coagulase-positive bacterium. *Staphylococcus aureus* infections have increased in recent years and are mainly associated with community-acquired methicillin-resistant *Staphylococcus aureus* (CA-MRSA). The morbidity of CA-MRSA doubled in the past decade [32]. CA-MRSA has a higher proportion of *Staphylococcus aureus* producing Panton-Valentine leucocidin (PVL) at 74–100%, which depends on the prevalence of methicillin-resistant *Staphylococcus aureus* (MRSA) in each region [33]. And there is no significant difference in PVL concentrations between PVL-positive methicillin-sensitive *Staphylococcus aureus* (MSSA) and PVL-positive MRSA [34]. PVL-positive strains are often associated with rapidly progressive, hemorrhagic NP [35].

PVL is a pore-forming toxin consisting of two components, LukS-PV and LukF-PV. It can form pores in the cell membrane of phagocytic leukocytes, especially polymorphonuclear leukocytes (PMNs), leading to leukocyte destruction and tissue necrosis [36]. Recombinant PVL is commonly used in experiments to explore the effects of PVL. In vitro, PVL was found to be a strong inducer for the release of histamine, proinflammatory mediators (IL-8, leukotriene-B4), granule enzymes (β-glucuronidase, hydrolase and lysozyme) and the production of reactive oxygen metabolites as a consequence of its effects in the activation and lysis of PMNs [36,37]. The increased influx of PMNs, infiltration of inflammatory cells, local vasodilation and tissue injury may result from this action. Similarly, PVL has been shown to induce the release of IL-8 in a rabbit model of NP [38]. Recombinant PVL can trigger the NOD-like receptor family pyrin domain containing 3 (NLRP3) inflammasome activation inducing macrophage death and IL-1𝛽 secretion [39]. Recombinant LukS-PV alone has been shown to induce the release of inflammatory cytokines from alveolar macrophages, and the toxicity of recombinant LukS-PV and LukF-PV has been found to form pores on the membrane of alveolar macrophages through binding to C5a receptor [40].

α-hemolysin is another critical virulence factor associated with NP. Purified α-hemolysin can establish a ferret NP model [41]. Like PVL, α-hemolysin can also activate NLRP3 inflammasome to induce IL-1𝛽 production and programmed necrotic cell death [42]. α-hemolysin can induce platelet-neutrophil aggregates formation, which plays an important role in alveolar capillary destruction in hemorrhagic/necrotizing pneumonia caused by CA-MRSA [43]. In fact, NP may be a shared consequence of numerous virulence factors like PVL, α-hemolysin, LukAB, Spa, toxic shock syndrome toxin-1, staphylococcal enterotoxin B and C, collagen adhesion, alpha toxin, staphylococcal enterotoxin-like toxin X, microbial surface components recognizing adhesive matrix molecules, clumping factors A and B, SdrE and the PSMs [44,45,46,47,48]. PVL promotes increased production of proinflammatory factor Spa, and they work together to cause overwhelming inflammation and tissue necrosis [48]. Clumping factors A and B, SdrE and Spa are able to activate platelet aggregation [49]. PVL also upregulates the expression of microbial surface components recognizing adhesive matrix molecules, which may enhance tissue adherence and colonization of PVL-positive stains [48]. Toxic shock syndrome toxin-1, staphylococcal enterotoxin B and C and staphylococcal enterotoxin-like toxin X are superantigens that have the ability to stimulate dysregulated T-cell proliferation [46]. *Staphylococcus aureus* can survive and proliferate within human cells free from the host immune system and antibiotic treatment [50]. Intracellular *Staphylococcus aureus* may cause apoptotic and/or necrotic cell death with several virulence factors involved by disturbing the host cell Ca^2+^ homeostasis and inducing cytoplasmic Ca^2+^ overload [51]. One possible mechanism is to create receptor-independent pore formation from the inner side of the plasma membrane [51]. For instance, α-hemolysin may induce Ca^2+^ permissive pores by nonspecifically integrating into membranes at high concentrations [52,53].

### 2.3. Mycoplasma pneumoniae

*Mycoplasma pneumoniae* is a common pathogen causing CAP in children and young adolescents. In some countries with PCV-13 included in national immunization programs, *Mycoplasma pneumoniae* has taken the leading role in pediatric CAP [54,55]. *Mycoplasma pneumoniae* is known as an “atypical” pathogen. In general, the clinical course of *Mycoplasma pneumoniae* pneumonia (MPP) is benign with high- or middle-grade fever and dry cough partly accompanied by chills, headaches and chest pain or tightness. In the past few years, there have been many reports of *Mycoplasma pneumoniae* NP in China and around the world [56,57]. One possible reason is that severe *Mycoplasma pneumoniae* pneumonia (SMPP) and refractory *Mycoplasma pneumoniae* pneumonia (RMPP) have been increasingly reported [58,59]. The appearance of macrolide resistance may have no effect on the disease severity, but delayed effective antimicrobial treatment can contribute to RMPP development and progress by leading to incapability to clear bacteria and excessive immune response [60,61]. In addition to the persistent infection, the older age of children with *Mycoplasma pneumoniae* NP may be associated with a strong immune response which is closely related to the severity of MPP [57]. Finally, severe bacterial or viral infections may follow or coincide with *Mycoplasma pneumoniae*, and NP may be the result of mixed infections [62,63]. Moreover, the early use of antibiotics makes the detection of coinfection with other pathogens more difficult.

Community-acquired respiratory distress syndrome (CARDS) toxin, membrane lipoproteins, hydrogen peroxide and superoxide are the main virulence factors of *Mycoplasma pneumoniae* [64]. CARDS toxin, the only exotoxin produced by *Mycoplasma pneumoniae*, possesses ADP-ribosyltransferase and cellular vacuolization properties [65]. ADP-ribosylation can induce inflammatory responses like activating NLRP3 inflammasome [66]. Thus CARDS toxin can cause cell swelling, nuclear lysis, cell vacuolization and ultimate cell death. Membrane lipoproteins, as lipopolysaccharides, can activate NLRP3 inflammasome and autophagy via TLR-4 [67]. Hydrogen peroxide and superoxide are both strong oxidants generated by *Mycoplasma pneumoniae* [68]. Moreover, superoxide has been reported to inhibit catalase activity [69]. Protein degradation and DNA lesions caused by hydroxylation and cell membrane damage caused by peroxidation of lipids may lead to cell death [70].

### 2.4. Co-Infection

Co-infection has the tendency to lead to severer pneumonia. In recent years, severe pediatric CAP caused by bacterial (pneumococcus, *Staphylococcus aureus*, *Pseudomonas aeruginosa*) or viral (adenovirus, rhinovirus, respiratory syncytial virus, parainfluenza virus, coronavirus, influenza virus, enterovirus, among others, respectively) coinfection with *Mycoplasma pneumoniae* has been increasingly reported [71,72,73]. NP associated with influenza virus or parainfluenza virus infections prior to pneumococcus or *Staphylococcus aureus* infections has been long reported [47,74,75,76,77,78]. Children co-infected with influenza A and *Staphylococcus aureus* are more likely to cause severe complications rapidly and have a higher mortality rate partly because of the difficulty of getting accurate and timely treatment [76].

Several hypothetical mechanisms of influenza virus during co-infection have been reported. Influenza virus can increase bacterial adhesion and growth by damaging lung epithelial cells, upregulating the host cell surface receptors and removing terminal sialic acid residues from host glycoconjugates [79,80]. Dysregulation of the immune system induced by the influenza virus may hinder the ability to clear the bacteria, for example, impairing the innate and adaptive immune responses by suppressing the Th1 immune cascade and the production of CD4- and B- lymphocytes and upregulating the production of T regulator cells [80,81]. The effect of PVL to damage the airway epithelium by killing neutrophils and the release of neutrophil proteases may be promoted by the influenza virus, which activates the lung epithelium to induce a massive influx of neutrophils. This activation of epithelium leads to the development of NP [82]. The influenza virus has been shown to modulate host metabolism, which is closely related to the levels of inflammatory cytokines expression and the promotion of influenza virus replication [83,84].

## 3. Clinical Manifestations

The common clinical manifestations of NP have no difference from those of uncomplicated pneumonia, including fever, cough, sometimes with chest pain, emesis, abdominal pain, dyspnea, anemia and fatigue [2]. But patients with NP have a more serious clinical course, longer duration of fever and length of stay, higher mortality, higher incidence of extra-pulmonary complications such as septic shock, respiratory failure and HUS and pulmonary complications such as parapneumonic effusion, pleural empyema, pyothorax, pneumothorax, pyopneumothorax and BPF [8,85]. PVL-positive *Staphylococcus aureus* is associated with high fever and an enhanced risk of purulent expectoration and hemoptysis [10]. And it has been shown that erythroderma, leucopenia and airway bleeding can predict the mortality of PVL-positive Staphylococcal NP [86]. Age and gender have no significant influence on the incidence of NP. The majority of reported cases are previously healthy and immunocompetent children [85]. When patients have flu-like symptoms, we should consider the possibility of viruses, especially during influenza season. When patients have malnutrition, cardiopulmonary diseases, underlying immunodeficiency conditions, or an experience of traveling or being in contact with birds or other animals, we should take the possibility of fungi into account [87]. Influenza-MRSA coinfection usually happens in previously healthy adolescents. They may have leukopenia or neutropenia and need ECMO support [88].

The laboratory characteristics of NP are mainly associated with mild-to-moderate anemia, hypoalbuminemia and elevated inflammatory markers, like white blood cell (WBC) counts and C-reactive protein (CRP) levels [2]. The typical pleural fluid of NP caused by bacteria has increased WBC counts, high protein content, low glucose concentration and high lactate dehydrogenase (LDH) concentration, while *Mycoplasma pneumoniae* NP is often dominated by lymphocytes, high protein content and normal glucose concentration in pleural fluid [4,89]. And organisms may be seen on Gram- or acid-fast-stained smears. The results of radiologic examinations could be large cavities and pleural effusions in the X-ray or on chest computed tomography (CT) [10].

## 4. Prediction and Early Recognition

In spite of the serious condition of NP, it always has a good prognosis with early recognition and management. Due to the variable evolution of NP, physicians’ awareness and experience, improved diagnostics, as well as the propensity of pathogens, is essential to predict the severity of the clinical course of CAP before deterioration happens.

### 4.1. Chest Computed Tomography and Lung Ultrasound

Diagnostic imaging is indispensable to NP. Chest radiography can reveal the presence of large cavities and large pleural effusions, but its accuracy does not compare with that of chest CT, especially in the initial phase of NP [90]. Ultrasonography is an effective modality to detect parenchymal lesions, pleural thickening and pleural effusion with no radiation exposure, low cost and wide availability [91]. And it has been shown to be as effective as CT in the diagnosis of NP and has been proposed to be used in the follow-up of imaging [92]. More importantly, impaired perfusion of Doppler ultrasound can predict massive necrotic changes early [93]. In the future, the use of chest CT in children with NP may be limited to particular occasions when patients do not respond well to appropriate management or are suspected of having complications or even other diseases. Currently, contrast-enhanced chest CT is still the most sensitive modality for the diagnosis of NP and can differentiate NP from a lung abscess [94]. It has been observed through CT scans that liquefaction transits to cavitation within 48 h [4]. In the lung consolidation area of NP, there are multiple thin-walled cavities or vesicles which can fuse into a large cavity. Compared with a single thick wall cavity of lung abscess, NP has no enhancement at the edge of the cavity. Some cavities have liquid levels and gas surfaces [94]. Pulmonary consolidations present several weeks before cavities appear. It has been shown that the CT value of pulmonary lesions in NP was lower than that in non-NP and may help predict NP early [95]. In order to reduce radiation damage, recent studies tried to adopt a machine-learning radiomics model based on radiographic features observed on non-enhanced CT scans or low-dose CT scans to recognize pulmonary consolidation in the early stage of NP in children [96,97]. Moreover, studies have found that lower kV combined with high Iterative Reconstruction in the CT pulmonary angiogram can maintain image quality [98,99].

### 4.2. Laboratory Data

The level of serum albumin has been extensively used to reflect the severity of patients’ conditions [100], and a recent study found hypoalbuminemia on admission was common in the pediatric intensive care unit and is a good predictor of mortality [101]. So it is meaningful to measure serum albumin routinely for NP patients. A retrospective observational study has shown that serum albumin less than 30.8 g/L is an independent risk factor for massive necrosis compared with mild and moderate necrosis [85]. WBC counts and CRP levels are common inflammatory markers. Increased WBC (≥15.1 × 10^9^/L) and CRP (≥121.5 mg/L) may have predictive significance for NP in children [102]. LDH, a pan-necrosis marker, can be released by cells undergoing either primary or secondary necrosis [103]. The level of pleural fluid LDH is often >1000 U/L in NP among children, while serum LDH is ≥353.5 U/L [2]. Thus, paying attention to the high level of LDH may be useful in informing clinicians of the possibility of ongoing necrosis or liquefaction of the pulmonary parenchyma [104]. In addition to these biomarkers that only predict the possibility of NP, some available biomarkers are expected to differentiate certain etiologies.

*Streptococcus pneumoniae* can produce neuraminidase which removes N-acetyl-neuraminic acid from cell membrane surfaces and exposes Thomsen-Friedenreich antigen (TA) present on erythrocytes, platelets and glomeruli [105]. The interaction between TA and anti-TA antibodies, called TA activation, can be assessed and might indicate a higher risk for NP [106]. Fetuin-A is a kind of sialoglycoprotein, and it can protect erythrocytes from TA exposure. The level of fetuin-A less than 340 mg/L might be a predictor for pneumococcal NP with or without HUS in children [107]. The presence of immature PMNs in peripheral blood, high CRP levels (>12 mg/dL) and no underlying disease at presentation have also been identified as independent predictors of the occurrence of necrosis or/and abscess caused by *Streptococcus pneumoniae* [5].

The critical factor of the severity of staphylococcal NP is PVL but not methicillin resistance [108]. Current approaches for detecting PVL, including enzyme-linked immunosorbent assay [34], latex agglutination [109] and polymerase chain reaction [110] are costly and time-consuming and are not routinely used at most diagnostic laboratories. Thus, it is promising to develop a rapid and affordable method for PVL detection, for instance, a novel lateral flow assay [111,112] and matrix-assisted laser desorption/ionization time-of-flight mass spectrometry detecting PVL within minutes [113]. And metabolomics may be a good way to predict illness severity prior to clinical diagnosis. For example, a study found the concentrations of acetone, acetoacetate, fumarate and glucose have been shown to be more than 25-fold higher in the patient with methicillin-resistant *Staphylococcus aureus* pneumonia than those of patients with influenza pneumonia and healthy controls [114]. But the overall concentration of the urine samples can be influenced greatly by the hydration state of the patient, so further investigations are needed.

D-dimer is a biomarker of fibrin formation and degradation and has been extensively used to diagnose venous thromboembolism [115]. Recently D-dimer levels have also been used to assess the severity of MPP [116], and D-dimer > 1367.5 ng/mL has been recognized as the risk factor for NP caused by *Mycoplasma pneumoniae* in children. And the use of low molecular-weight heparin has been shown to reduce the risk of pulmonary necrosis [117]. Children with *Mycoplasma pneumoniae* pneumonia (MPP) infection are related to higher risks of blood coagulation and thrombosis. Several studies have shown that pulmonary embolism, stroke, splenic infarction, myocardial infarction and even extensive thrombotic events are associated with *Mycoplasma pneumoniae* infection in children [118,119,120,121,122]. Furthermore, a recent study demonstrates that elevated D-dimer, specifically >11.1 mg/L (even >5.0 mg/L), would assist in the early diagnosis of thrombosis in MPP [123]. However, another recent study has shown that there is no statistically significant difference in D-dimer between *Mycoplasma pneumoniae* NP and non-*Mycoplasma pneumoniae* NP and even no case of pulmonary embolism was observed in NP [57]. On the other hand, higher levels of IL-6 and IgE were also observed in NP caused by MPP compared with non-NP [95,124]. And CT value of large pulmonary lesions ≤ 36.43 and IFN-γ ≤ 7.25 pg/mL might help us to early predict NP from MPP with large pulmonary lesions in children [95].

Clinically, it is necessary to be vigilant about the possibility of NP in previously healthy and immunocompetent children with lobar pneumonia, especially when clinical manifestations, radiographic features and laboratory data like reduced serum albumin, elevated inflammatory markers and increased D-dimer do not improve or even worsen in spite of antibiotics with supportive care. If radiographic features reveal pulmonary consolidations and/or the CT value of large pulmonary lesions is ≤36.43 and/or laboratory data exceeds a certain range (WBC ≥ 15.1 × 10^9^/L, CRP ≥ 121.5 mg/L, D-dimer > 11.1 mg/L, IFN-γ ≤ 7.25 pg/mL, serum LDH ≥ 353.5 U/L and/or pleural fluid LDH > 1000 U/L), NP would be more suspicious. Although culture is time-consuming, analyzing drug sensitivity is still the most important thing in treating bacterial infections. In the meantime, current molecular biology methods make it possible to identify the responsible pathogen rapidly. The combination of laboratory data and certain pathogens identification like MASA and refractory *Mycoplasma pneumoniae* would help diagnose NP.

## 5. Management

### 5.1. Antibiotics and Adjunctive Therapy

To date, recommendations for the optimal management of NP rely mainly on expert opinion for the lack of high-quality randomized controlled trials. In general, the initial treatment is conservative in immunocompetent children. Intravenous (IV) antibiotics with supportive care are the mainstay of therapy. The regimen for NP needs to contain both anti-pneumococcal and anti-staphylococcal antibiotics, such as high-dose penicillin or ampicillin, amoxicillin-clavulanic acid, or second-generation or third-generation cephalosporin [1]. When coinfection with *Mycoplasma pneumoniae* is documented, a macrolide like IV azithromycin should be included in the treatment [63]. Local epidemiologic and patterns of antibiotic resistance should be taken into consideration. In areas where multidrug-resistant pneumococcus or MRSA is prevalent, vancomycin is usually added as the first-line agent before the availability of culture results [125,126]. However, a recent study showed empiric vancomycin for children predicted mortality and poor outcomes [126]. An increased risk of nephrotoxicity has been reported in several pediatric studies [126,127]. It is difficult to reach therapeutic levels of vancomycin in children with good renal clearance, and vancomycin can penetrate poorly into lung tissues [88]. Protein synthesis inhibitors such as linezolid, clindamycin, or rifampicin may be a better alternative to vancomycin for the treatment of NP caused by PVL-positive *Staphylococcus aureus*, but their clinical evaluation is not available [128]. Antimicrobial peptides have been a hotspot of multidrug-resistant bacteria. It has been reported that ɑ-defensins can neutralize PVL and inhibit its cytotoxicity [129]. Antimicrobial peptides may be a new kind of antibiotic for NP in the future. If patients do not respond well, treatment can be tailed according to the results of antibiotic susceptibility testing [125].

For NP caused by SMPP or RMPP, the combination of macrolide and systemic corticosteroids may be an effective treatment [130]. However, there is a lack of studies to determine its effectiveness and safety. IV immunoglobulin containing pooled human polyclonal antibodies can not only modulate the immune system but also neutralize specific lung-damaging toxins. It could be considered an adjunctive therapy, but clinical studies are still rare [128,131]. To eliminate intracellular MASA, an antibody-antibiotic conjugate consisting of an anti-*Staphylococcus aureus* antibody and the rifamycin derivative rifalogue may be a novel way [132]. When *Mycobacterium tuberculosis*, viruses, or fungi are suspected, anti-tuberculosis, anti-viral, or anti-fungal therapy should be added, respectively.

### 5.2. Flexible Bronchoscopy and Surgery

Flexible fiberoptic bronchoscopy with bronchoalveolar lavage is an important modality of diagnosis and therapy in pediatric patients [133]. It can detect endobronchial abnormalities, obtain more efficient samples for bacteriologic, cytologic and histologic detection and offer therapeutic interventions [134]. Taking the opportunity to apply bronchoscopy has been shown to promote rapid recovery in refractory pneumonia, while bronchoscopy for NP has been rarely reported [76,133,135]. Chest drains, a kind of interventional procedure, are used to remove large empyema or parapneumonic effusion or pyopneumothorax in children with NP. It has been reported to have similar outcomes treating NP with surgical treatments [4]. Surgical treatments of pediatric NP are recommended in cases where there is the presence of complications or no complete response to the conservative treatment. A study found that a delay in surgery is associated with more complications and recommended an early intervention [136]. Early thoracoscopy may hasten recovery and avoid late lung resections [137]. However, postoperative complications such as BPF or small pneumatoceles are common, and surgical treatments are associated with wound infections, bleeding, prolonged pain and recovery times [136,138]. In fact, lung resection involving wedge resection or pneumonectomy in children is rare because it may impair future pulmonary function in spite of having little effect on postoperative FVC or FEV1 [139]. Most pneumatoceles can resolve spontaneously within weeks or months, while 80% of BPF in children need surgical treatments [30,140]. When fever and complications like acute respiratory failure, BPF, empyema and sepsis persist, video-assisted thoracoscopic surgery or mini-thoracotomy may be required [141]. Thoracotomy can debride fibrinous material, excise necrotic tissue, close air leaks and drain pus from the pleural cavity [137]. Endobronchial embolization, which is mainly used in adults, may be a less invasive alternative treatment for large pneumatoceles and BPF in children than the classic surgical approach [140].

## 6. Conclusions

*Streptococcus pneumoniae*, *Staphylococcus aureus* and *Mycoplasma pneumoniae* are the main infectious agents causing NP. Although detailed mechanisms are still not clear, we expect to recognize them earlier, take timely treatment measures and develop novel therapeutic strategies. Current studies focus on radiological outcomes, clinical features, laboratory characteristics and the development of testing technology. In the future, predictors from these aspects may be used in clinical. 

## Figures and Tables

**Table 1:** Prevalent serotypes of *Streptococcus pneumoniae* in NP.

Year	Country	No.	PCV Use in National Immunity Program	Prevalent Serotypes Pre-PCV	Prevalent Serotypes Post-PCV	PCV Coverage	References
1997–2006	Utah	33	PCV-7: 2001, 2 + 1	Pre-PCV7: 6B (40.0%), 19F (20.0%), 1 (20.0%) and 6A (20.0%)	Post-PCV7: 4 (3.6%), 1 (7.1%), 3 (39.3%), 7 (3.6%), 8 (3.6%), 17 (3.6%), 19 (14.3%) and 19A (14.3%)	PCV-7: 80%	[20]
2005–2019	Italy	43	PCV-13: 2010, 2 + 1	Pre-PCV13: 1 (18.2%), 3 (27.3%), 5 (9.1%), 7F (9.1%) and 19A (9.1%)	Early post-PCV13: 1 (25.0%), 5 (12.5%) and 7F (12.5%) Late post-PCV13: 3 (80.0%) and 12 (20.0%)	PCV-7: 62.8% PCV-13: 23.3%	[23]
2012–2016	Catalonia, Spain	35	PCV-13: 2016, 2 + 1	Not mentioned	Post-PCV13: 1 (14.3%), 3 (48.6%), 6B (2.9%), 7F (2.9%), 14 (5.7%), 19A (8.6%), 7A (5.7%), 6A/C (2.9%) and 12F/A/44/46 (2.9%)	PCV-13: 14.3%	[24]

## Data Availability

Not applicable.
